# Effect of Alcalase Modification of Yellow Lupin (*Lupinus luteus L.*) Protein Isolate on Some Functional Properties and Antioxidant Activity

**DOI:** 10.1155/2022/6187441

**Published:** 2022-11-18

**Authors:** Iwona Tesarowicz, Agnieszka Zawiślak, Ireneusz Maciejaszek, Krzysztof Surówka

**Affiliations:** Department of Biotechnology and General Food Technology, Faculty of Food Technology, University of Agriculture in Krakow, Al. Mickiewicza 21, 31-120 Kraków, Poland

## Abstract

Protein isolate obtained from sweet yellow lupin (*Lupinus luteus L.*) and its Alcalase hydrolysates were examined for their functional and antioxidant properties in relation to surface hydrophobicity of proteins and peptides and molecular weight distribution. Enzymatic hydrolysis improved the foaming characteristics of lupin proteins, while the emulsifying properties deteriorated. It means that good foaming properties of preparations are determined by the presence of low-molecular *δ* conglutin and small subunits of *γ* conglutins. In turn, larger proteins such as *α* and *β* conglutin are responsible for maintaining good emulsifying properties. The measured surface hydrophobicity was consistent with the results of emulsifying properties. It has also been shown that the scope of changes in antioxidant properties due to hydrolysis, measured by DPPH method and as reducing power, is more pronounced than with the use of ABTS and FRAP methods.

## 1. Introduction

The use of legume seeds as a source of food protein is of growing interest to consumers, especially in view of the increasing trend towards vegetarian diet. From a global perspective, soybeans are traditionally the most popular protein raw material from this group of plants, although consumers also appreciate legumes such as chickpea, pea, and lentils.

Relatively less attention is paid to lupin, which is after all a valuable legume with high protein content, comparable with that in soy. The protein quality of its seeds is equally good and rated by some authors as better than soybeans. Although it is poor in sulfur-containing amino acids, it also has less antinutritional factors than soybeans—trypsin inhibitors and hemagglutinins are practically absent [1–3].

Lupin is also easy to grow because it tolerates frost, drought, and poor soils quite well. However, although known as human food since early Roman times, it is utilized to a small extent so far, primarily because of alkaloid content and low agronomic yield. Commonly found in lupins are quinolizidine alkaloids such as lupanine, sparteine, or lupinine, which have been shown to be reversible inhibitors of acetylcholinesterase. Alkaloid level depends on cultivar, soil type, and growing season. Some cultivars have been developed, however, which are sweet and contain alkaloids at the level safe for human health [2, 4, 5]. Moreover, alkaloid content can be substantially decreased by soaking and rinsing lupin beans as well as by technological processing.

It is believed that lupin can be widely used as a good source of protein, fiber, and as a functional supplement in many food products. Lupin flour, containing almost 40% of protein, is a good raw material for obtaining protein preparations. In the available literature, only limited number of reports could be found about the use of enzyme proteolysis to modify functional and antioxidant properties of lupin proteins [6–8]. Functional properties of lupin protein are considered as relatively good [2, 3]. However, they can be modified in order to change, for example, their foaming and emulsifying ability or other features affecting their technological suitability. An important and widely approved method of modifying vegetable proteins is enzymatic hydrolysis. Due to this method, Lqari et al. [6] improved some functional properties of *Lupinus angustifolius* protein. In turn, Surówka et al. [9] showed that limited enzyme proteolysis enables the improvement of the emulsifying and foaming properties of extruded soy flour.

It was assumed that limited enzymatic hydrolysis of proteins included in the lupin protein isolate (LPI) will not only allow for favourable modification of their technological properties but will also affect antioxidant capacities. Therefore, the aim of this study was to produce the LPI, carry out its proteolysis with Alcalase, and to check whether the hydrolysates with the desired foaming and emulsifying and health-promoting properties are produced under the applied process conditions. The hydrolysates obtained may potentially be used as functional ingredients protecting food products from oxidative deterioration.

## 2. Materials and Methods

### 2.1. Materials

**Figure 1:**
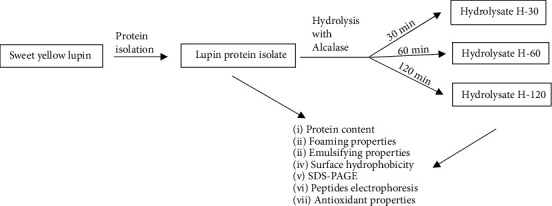
Flowchart presenting the experimental design.

Seeds of sweet yellow lupin (*Lupinus luteus L.* var. Mister) were obtained from Poznańska Hodowla Roślin Ltd, Plant Breeding Station Wiatrowo (52°45' N; 17°08' E). They served as a raw material for obtaining LPI and protein hydrolysates according to the flowchart shown in Figure 1.

Food grade alkaline bacterial protease Alcalase of *Bacillus licheniformis* (Novo Nordisk A/S) with activity equal to 2.4 Anson units/g was used for hydrolysis.

### 2.2. Protein Isolation

Lupin beans were ground in Thermomix (Thermomix 31-1, Vorwerk, Germany) and sieved to collect the fraction 250 *μ*m of lupin flour, which was then used in further experiments. In order to obtain lupin protein isolate (LPI), 10% suspension of lupin flour (LF) in deionized water with the addition of NaHSO_3_ (0.77 mM) was brought to pH 8.6 with 1.0 M NaOH and stirred for 40 min at 30°C [10]. Subsequently, suspension was centrifuged at 2000 × g for 20 min, and protein was precipitated from supernatant by acidification to pH 4.5 with 1.0 M HCl. Whole was stirred for 15 min, then centrifuged for 20 min (900 × g). Protein precipitate was rinsed twice with water, decanted, suspended in deionized water in a ratio of 1 : 9, neutralized with 1.0 M NaOH, and lyophilized.

### 2.3. Hydrolysis

Lyophilized LPI was subjected to enzymatic hydrolysis. For this purpose, 25 g sample was suspended in 225 mL of deionized water, and Alcalase solution was added to obtain enzyme : substrate ratio 18 mAU/g protein. Hydrolysis was carried out at 55°C, and pH 8.5 was kept constant with 1.0 M NaOH. As a result, three hydrolysates were obtained, which differed in processing time (0.5 h, 1 h, and 2 h; for hydrolysates H-30, H-60, and H-120, respectively).

The process was terminated by heating reaction environment for 15 min in 90°C. Subsequently, samples were cooled and centrifuged (15 min, 4000 × g). Supernatants were frozen and lyophilized.

### 2.4. Proximate Composition

The amount of protein (*N* × 6.25) was detected by means of the Kjeldahl method using K-435 and B-324 combustion and distillation units, respectively (Büchi, Flawil, Switzerland) [11].

### 2.5. Foaming and Emulsifying Properties

Foaming properties were studied according to the method described by Surówka et al. [9]. Foam was created by passing argon at a rate of 10.4 mL/s through 100 mL of analysed hydrolysate solution (2% total protein). Following parameters were determined: foaming capacity (FC), foam overrun (FO), and foam stability as a liquid drainage (LD_5_). Foaming capacity was calculated as the ratio of foam volume to the volume of pressed gas necessary to its production. Foam overrun was constituted by ratio of the gas volume in the foam to the volume of the liquid involved in its formation. In turn, liquid drainage representing foam stability was the ratio of the volume of foam liquid released during 5 min to the volume of liquid in the foam at the moment of finishing the aeration [12–14].

Emulsifying activity (EAI) and emulsion stability (ESI) indexes were analysed turbidimetrically [9, 15, 16]. EAI informs about the oil/water interface area (m^2^), which can be formed by 1 g protein of the analysed hydrolysate in the experimental conditions, whereas ESI was calculated from the formula:

(1) $$\begin{array}{c} \text{ESI}=\frac{A_{0}\times 5\min}{A_{0}-A_{5}}, \end{array}$$

where *A*_0_–turbidance measured at 500 nm immediately after homogenization and *A*_5_–turbidance measured at 500 nm after 5 min after homogenization.

### 2.6. Surface Hydrophobicity

Surface hydrophobicity was determined by the spectrofluorimetric method of Hayakawa and Nakai [17] with the use of 1-anilino-8-naphthalene-sulfonate (ANS) as the fluorescence probe. Fluorescence intensity (FI) was measured for two dilution series of the examined sample solutions (0.03-0.8 mg protein/mL), one was without ANS and the other with ANS (8 mM ANS). Measurements were performed using a Cary-Eclipse spectrofluorimeter, at wavelengths *λ*_ex_ and *λ*_em_ 390 and 470 nm, respectively. The Netto value of fluorescence intensity (FI_NETTO_) was calculated by subtracting FI of each solution without probe from that with probe. The plot of FI_NETTO_ versus protein concentration leads to determination of slope, which was established as an index of the surface hydrophobicity.

### 2.7. Electrophoretic Separations

To analyse molecular weight distribution of protein, lyophilized samples were homogenized in distilled water to obtain 1% protein suspensions. The aliquots of homogenate were diluted (1 : 1) with a denaturing buffer (0.125 M Tris, 4% SDS, 20% glycerol, 2% 2-mercaptoethanol, and pH 6.8) and heated for 90 s in a boiling water bath. The extracts were centrifuged at 5000 g for 15 min, and clear supernatants were collected. Sodium dodecyl sulfate-polyacrylamide gel electrophoresis (SDS-PAGE) was carried out according to the Laemmli [18] method using a 12.5% w/v gel concentration. The molecular weights of the bands were estimated using MW-SDS-200 and MW-SDS-70 L marker kits (Sigma Chemical Co., St. Louis, MO, USA). 0.025% Coomassie blue R-250 solution in 40% methanol and 7% acetic acid was used for staining of gels. Destaining was carried out with the use of methanol/acetic acid solution (40% v/v; 7% v/v), which was changed until a colourless gel background was obtained. For peptide separations, Schägger and von Jagow's [19] procedure was applied. The separation was carried out in a discontinuous system in the presence of a buffer containing tricine. A Hoefer Mighty Small SE 260 unit coupled with an EPS 301 power supply unit (Amersham Pharmacia Biotech, Uppsala, Sweden) was employed in all electrophoretical analyses. Stained gels were scanned and analysed using Image Master TotalLab (Amersham Pharmacia Biotech, Uppsala, Sweden) software.

### 2.8. Antioxidant Properties

Antioxidant activity was measured using four different spectrophotometric techniques [20]. Two radical scavenging methods were employed, one with the use of 2,2-diphenyl-1-picrylhydrazyl (DPPH) according to Brand-William's method [21] and the other applying 2,2'-azino-bis (3-ethylbenzothiazoline-6-sulfonic) (ABTS) in accordance with Re et al. [22]. Two remaining analyses were based on the reduction of Fe^3+^ ions to Fe^2+^ ions, resulting in the formation of intense blue colour. In the Benzie and Strain method [23], the ions are complexed by 2,4,6-tris (2-pyridyl) -1,3,5-triazine (TPTZ). In the Yen and Chen method [24], the solution of prussian blue forming during reduction reaction is responsible for sample colour.

Aqueous extracts of the hydrolysate and isolate were prepared for the assays. For this purpose, 2.5 g of relevant lyophilisate was mixed with 47.5 mL of deionised water. The 5% extracts were used for further analyses.

#### 2.8.1. Determination of Antioxidant Activity Using DPPH Free Radicals

The volume of 1 mL of the extract and 4 mL of DPPH solution (0.0394 g/L ethanol) was mixed. The absorbance was measured immediately after mixing at a wavelength of 517 nm relative to pure ethanol. The next measurement was performed after half an hour. The amount of DPPH free radicals remaining in the reaction mixture after 30 minutes was calculated as

(2) $$\begin{array}{c} \%\text{DPPH}=100*\frac{\left[{\left(A_{517}\right)}_{T_{0}}–{\left(A_{517}\right)}_{T}\right]}{\left[{\left(A_{517}\right)}_{T_{0}}\right]}, \end{array}$$

where (*A*_517_)_*T*_ is the mixture absorbance at the end of the experiment and (*A*_517_)_*T*0_ is the absorbance of the mixture at the beginning of the experiment.

#### 2.8.2. Determination of Antioxidant Activity Using ABTS Free Radicals

The ABTS radical cations were previously prepared from the 2,2'-azino-bis (3-ethylbenzothiazoline-6-sulfonic acid ammonium salt) by oxidation with sodium persulfate. Firstly, a stock solution was prepared by dissolving 0.0960 g ABTS and 0.0165 g of sodium persulfate in 25 mL of distilled water. Subsequently, after 24 hours in the dark, 1 mL was taken from the stock solution and dissolved in 50 mL of PBS (phosphate buffer) solution. The assay was carried out by taking 2 mL of the initial solution and 1 mL of extract. The whole was stirred, and after 10 minutes, the absorbance was measured at a wavelength of 734 nm against a PBS solution. A reference test was also performed; the measurement was taken directly after the addition of free radicals, in which the extract was replaced with a PBS solution. The rate of reduction of the radicals (% RSA) was calculated according to the formula:

(3) $$\begin{array}{c} \%\text{RSA}=\frac{\left[\left(E_{0}-E_{10}\right)\right]}{\left[E_{0}\right]}*100, \end{array}$$

where *E*_0_ is the absorbance of the reaction mixture at the beginning of the analysis and *E*_10_ is the absorbance of the reaction mixture at the end of the determination.

#### 2.8.3. Determination of Antioxidant Activity by the FRAP Method

Acetate buffer (300 mM, pH 3.6), FeCl_3_ (20 mM), and TPTZ solution (8 mM) were mixed in hydrochloric acid (40 mM) in a 10 : 1 : 1 ratio to prepare working reaction mixture. An amount of 0.4 mL of the extract and 3.6 mL of the TPTZ working solution was mixed and incubated for 10 min at 37°C. Subsequently, the solution was centrifuged for 2 min at 4000 rpm. The absorbance of supernatant was measured at 595 nm against a blank, in which the extract was substituted with distilled water. The results were expressed in trolox equivalents (mmol/10 mL).

#### 2.8.4. Determination of Reducing Power

The volume of 1 mL of extract, 2.5 mL of phosphate buffer (0.2 M, pH 6.6), and 2.5 mL of potassium ferrocyanide (K_3_[Fe (CN)_6_]; 10 g/L) was mixed, and the whole was incubated at 50°C for 30 min. Subsequently, 2.5 mL of trichloroacetic acid solution (100 g/L) was added to the mixture and vortexed. Afterward, the entire mixture was centrifuged at 4500 rpm for 15 min. The supernatant (2.5 mL) was transferred to a tube, and 2.5 mL of distilled water was added along with 0.5 mL of iron (III) chloride solution (1 g/L). After 7 minutes, the absorbance at 700 nm was measured, relative to a blank in which the extract was replaced with distilled water. Results were converted to ascorbic acid equivalents (mg/100 mL), based on the standard curve.

### 2.9. Statistical Analysis

All experiments except for electrophoresis were carried out in triplicate. Results of measurements were statistically analysed by analysis of variance (ANOVA) using the CSS Statistica v. 12 package (StatSoft, Kraków, Poland). Differences were considered significant at *P* < 0.05 using Duncan multiple range test. Data were presented as means ± standard deviation (SD).

## 3. Results and Discussion

### 3.1. Protein Content, Molecular Weight Distribution, and Surface Hydrophobicity

In vegetable protein products protein concentration, molecular weight distribution and surface hydrophobicity determine their functional properties.

In this study, lupin protein isolate, with 87.9 ± 0.2% protein content, was produced from yellow lupin flour containing 39.3 ± 2.4% protein, by means of alkaline leaching and isoelectric precipitation. El Adawy et al. [3] reported higher protein content in the isolate (about 91%); however, process parameters applied by them were slightly different. In turn, Lampart-Szczapa and Mossor [25] received an isolate containing approximately 4% less of protein. The enzymatic hydrolysis has a little effect on the content of protein substances. In the H-30, H-60, and H-120 hydrolysates, they were found at the level of 89.4 ± 2.0, 90.1 ± 1.1, and 87.4 ± 0.3%, respectively, and were statistically different than in the isolate (*P* < 0.05). Schlegel et al. [8] did not observe a significant change in protein content due to enzymatic proteolysis of lupin protein isolate.

**Figure 2:**
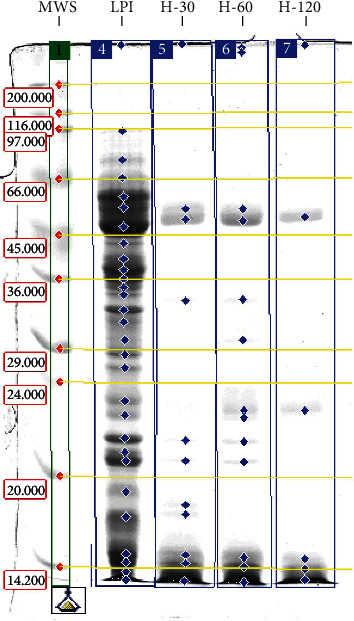
The results of SDS-PAGE of lupin protein preparations. Lanes: MWS: molecular weight standards (Da); LPI: lupin protein isolate; H-30, H-60, and H-120: LPI hydrolysates.

The quantitative results of SDS-PAGE separation of proteins in LPI and hydrolysates are shown in Figure 2 and Table 1. According to Capraro et al. [26] and Duranti et al. [27], 7S-*β*- and 11S-*α*-globulins are predominant in lupin flour proteins. This corresponds to the SDS-PAGE results, since the proportion of protein fractions of 42-66 kDa was the highest (30.7%) in the analysed nonhydrolyzed LPI. It is assumed that within this range, there are subunits of one of the two main globulin fractions: legumin-like *α* conglutins (11S-*α*-globulins with above 330 kDa molecular weight) and vicilin-like *β* conglutins (7S-*β*-globulins with molecular weight of 143-260 kDa). Legumins are hexamers composed of heterogeneous acidic subunits (42-52 kDa) and basic subunits (20-22 kDa), while vicilins are trimers consisting of three subunits, which molecular weights range between 17 and 64 kDa (HMW: 53-64 kDa, IMW: 25-46 kDa, and LMW: 17-20 kDa) [27]. Since these fractions contain polypeptides with similar molecular weights, it is not possible to distinguish these subunits clearly on the polyacrylamide gel used.

The next important protein fractions of the LPI (17%) were low-molecular-weight proteins below 15 kDa. These proteins are probably part of the *δ* conglutins (2S globulins) with molecular weight of about 13 kDa, composed of subunits: small (4 kDa) and large (9 kDa) [27]. In this work, the fraction of proteins in the molecular weight range of 34-40 kDa was represented by a fairly distinct band, representing 16% of all proteins in LPI. It is, most probably, a protein identified in the literature as an IMW subunit of *β* conglutins [26, 28]. A band with a MW in the range of 20-22 kDa had a similar relative share in LPI proteins representing basic subunits of legumins.

Analysis of the obtained electrophoretic data showed that some fractions were occurring only in individual lines, and there were others, which repeated in all samples–namely in LPI and hydrolysates. The repetitive fractions visible on the gel scan (Figure 2) were mainly proteins with molecular weights 42-66 kDa, partly 26-34 kDa, 20-22 kDa, and low-molecular proteins (<15 kDa).

In hydrolysates, the main fractions (above 70%) were proteins with a molecular weight below 15 kDa. The relative content of these low-molecular-weight fractions in enzyme hydrolysates was 4-5 times higher than their level in the raw material (LPI). Similar results of the hydrolysis with Alcalase were obtained by Schlegel et al. [8] who, as a result of proteolysis, received mainly peptides with molecular weights below 23 kDa. The authors carried out a more advanced LPI hydrolysis of *Lupinus angustifolius L.* in order to reduce the abundance of major allergens and concluded that Alcalase was effective in the hydrolysis of the high-molecular-weight fractions of *α* conglutin and *β* conglutin, as well as the medium molecular weight fractions of LPI.

In our study, when comparing the LPI and products of its modification with Alcalase, a significant decrease in the content of the high-molecular-weight (HMW) *β* conglutin (53-66 kDa) and acidic *α* conglutin (42-52 kDa) subunits, as well as in other polypeptide chains of molecular weight above 15 kDa, was observed. This is due to the breakdown of proteins into low-molecular-weight peptides and amino acids. However, compared to other bands, the loss of the fractions 42-66 kDa and 20-22 kDa is relatively less than, for example, fractions with molecular weights in the 34-40 kDa, 26-34 kDa, or 18-20 kDa range. This suggests a greater susceptibility of IMW and LMW subunits of *β* conglutin and large subunits of *γ*-conglutin to the proteolytic activity of Alcalase in comparison to the other fractions of LPI. Present after hydrolysis with Alcalase but faintly marked bands of about 20-22 kDa, probably originated from 11S globulins, were also observed by Karamać et al. [29], who studied flaxseed protein hydrolysates, as well as by Wang et al. [30], who studied soybean meal protein hydrolysates. The authors of the second work pointed the difference of various soy proteins in sensitivity to different proteolytic enzymes. As they indicate, the acidic subunits of glycinin (11S protein) are easier to be hydrolyzed than basic subunits, which in turn contain more hydrophobic amino acids and are rather located in the interior of the structure of glycinin.

Analysis of protein band percentages showed differences between individual hydrolysates, which may affect differences in the examined functional properties. The product obtained as a result of two-hour proteolysis (H-120) had higher content of low-molecular-weight protein fractions (below 15 kDa) than H-30 and H-60 hydrolysates.

**Figure 3:**
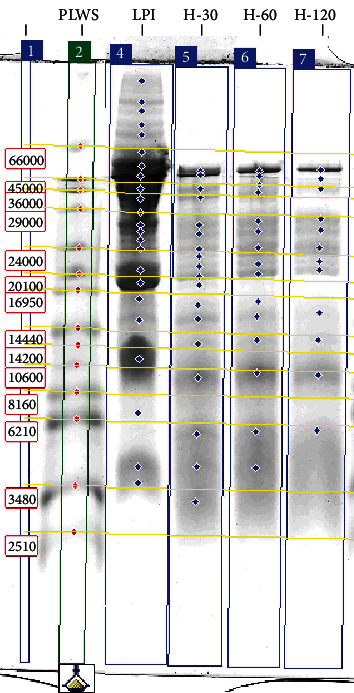
Results of the peptide separation (Schägger and von Jagow [19] method) of lupin protein preparations. Lanes: PLWS: peptide and low-molecular-weight standards; LPI: lupin protein isolate; H-30, H-60, and H-120: LPI hydrolysates.

In order to identify the products of proteolysis more accurately, the separation of peptides was performed according to the Schägger and von Jagow procedure [19], using a tricine cathode buffer. The results of the peptide separation are given in Figure 3 and Table 2.

The electrophoretic analysis by means of the Schägger and von Jagow procedure revealed that proteins with molecular weights in the range of 43-65 kDa (more than 26%) were predominant in LPI, and this result confirmed the findings obtained by the standard SDS-PAGE electrophoresis. With regard to hydrolysates, however, the predominant protein fraction changed with a prolongation of the proteolysis length. It was found that in hydrolysates H-30, H-60, and H-120, the dominant compounds were those with molecular weight of 43-65 kDa, 24-33 kDa, and below 10 kDa, respectively. The bands that represented the low-molecular-weight fractions in these preparations were wide and fuzzy. The observed phenomenon is a result of polydispersity, i.e., an approximately continuous molecular weight distribution, with no dominant bands corresponding to a higher content of the molecular weight fraction.

**Figure 4:**
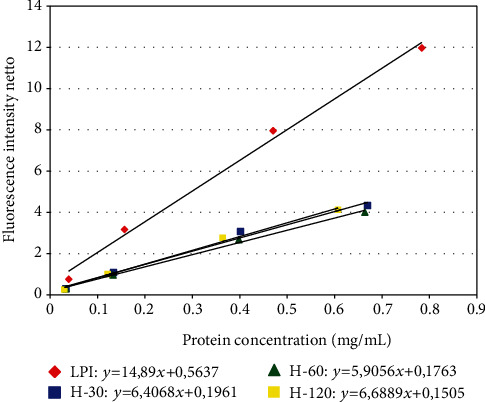
Surface hydrophobicity analysis results of LPI and its hydrolysates (H-30, H-60, and H-120).

The results obtained for the surface hydrophobicity of lupin protein preparations, presented as a plot and the simple equations fitted to them, are shown in Figure 4. The highest value, expressed as a slope in a straight line equation representing changes in fluorescence intensity with increasing protein concentration, was observed for the LPI and amounted to 14.89. In turn, the hydrophobicity of hydrolysates was distinctly lower (*P* < 0.05) and assumed similar values to each other (5.90–6.69), showing no statistically significant differences (*P* > 0.05). This indicates that the initial hydrolysis of lupin proteins is already enough to reduce this parameter significantly; the prolongation of the process does not change it substantially.

Similar results of reducing surface hydrophobicity were obtained by Surówka et al. [31], who hydrolyzed soy protein concentrate and its extrudate using both Alcalase and Esperase. A decrease in surface hydrophobicity as a result of proteolysis with Alkalase was also observed during porcine plasma protein hydrolysis [32]. As the authors of above works point out, the change in hydrophobicity—its increase or decrease—as a result of proteolysis is associated with the shortening of peptide chains and depends mainly on the nature of the hydrolyzed protein and molecular weight size of the formed peptides. As in the aforementioned studies, the decrease in surface hydrophobicity of the hydrolysates obtained in this study may be explained by the fact that the released peptides from the native structure of protein demonstrate great flexibility, which enables a conformation with hydrophilic moieties more exposed outward. The obvious consequence of changes in surface hydrophobicity is a change of interfacial functional properties, such as foaming and emulsifying properties.

### 3.2. Functional Properties

Table 3 shows the results of the functional properties analysis of investigated preparations. The foam formed due to aeration of the LPI solution was quite difficult to obtain and was characterized by a coarse structure and very thin walls of the film. It was, however, relatively stable. The values of FC, which when higher reflect better foaming efficiency, increased noticeably as a result of hydrolysis. This index in all tested hydrolysates was about 10% higher than in the LPI. This means that the process of proteolysis significantly improves the foaming properties.

In foams characterized by high FO values, gas bubbles are large, and/or the foam walls are relatively thin. Thus, high FO values indicate a technologically unfavourable structure of the foam. As for hydrolysate preparations, the FO values decreased significantly compared to the FO values determined for the LPI. As a result, the foam obtained from the hydrolysate solutions had a desirable, more finely porous structure with strong walls. As in the case of FC, an improvement of this aeration property was noted due to enzymatic hydrolysis. However, according to the data in Table 3, the LD_5_ value increased, which means that foam stability decreased as compared to the LPI. Moreover, this decrease seems to be to some extent related to the length of the process. Therefore, in order to improve the foam stability of hydrolysate solutions, they should be used in preparations with stabilizing substances, e.g., polysaccharides increasing the viscosity.

According to Surówka et al. [31], who investigated soy protein hydrolysates, and Lqari et al. [6], who examined narrow-leafed lupin, partial enzymatic hydrolysis improves the foaming capacity compared to the raw material, from which the hydrolysate was prepared. However, the foams from these hydrolysates were not very stable. This agrees with our results obtained for the lupin hydrolysates, according to which peptides and *δ* conglutins, the LMW subunits and small subunits of *β* and *γ* conglutins had better adsorption capacity on the surface of air bubbles, providing good foaming properties to these products. Peptides present in hydrolysates can reach the interfacial surface much faster than proteins and create films that form the foam structure. The above authors concluded that degree of hydrolysis determines the foaming capacity of hydrolysates; with an increase in the degree of hydrolysis and a decrease in the average molecular weight, the ability to form foams increases. This is due to the fact that proteins with lower molecular weight have a greater ability to adsorb on the surface of air bubbles. Polar moieties located on the surface of the protein molecule more easily turn towards liquid and nonpolar ones towards the air. As a result, a coherent, flexible film forms around the air bubbles.

Emulsion with the use of LPI is formed relatively efficiently as measured by EAI (Table 3). This index can be slightly improved by applying mild hydrolysis conditions, as was the case with limited enzymic hydrolysis of soy flour extrudates [9]. In this study, however, such improvement was not observed. The hydrolysate obtained after the half-hour process (H-30) produced emulsion with EAI lower by 22% compared to the isolate. Prolongation of the hydrolysis resulted in hydrolysates having even lower EAI values.

Alcalase hydrolysis had an even greater effect on ESI. While the emulsions obtained from LPI showed good stability, as a result of the hydrolysis, the ESI index decreased several times (Table 3).

It is believed that the surface hydrophobicity of proteins affects the emulsifying properties [33, 34]. When comparing the results of the analysis of emulsifying properties and surface hydrophobicity of LPI and its hydrolysates, it can be approximated that the results of hydrophobicity analysis and EAI and ESI are convergent, i.e., the hydrolysis leads to a deterioration of these indexes in line with the decrease of hydrophobicity.

In this study, as a result of *α* and *β* conglutins fragmentation, the number of peptides increased, but the number of both hydrophobic and hydrophilic moieties in their molecules was reduced. Even when the peptides are positioned on the surface of the dispersed lipids, interactions with the aqueous phase, which stabilize emulsions, will not be formed. In consequence, a loss is observed of emulsifying properties and decreased stabilization of the emulsion. Therefore, among the analysed preparations, LPI showed better emulsifying properties than hydrolysates. Similar observations were reported in studies on rape protein hydrolyzed with Alcalase, where a decrease in emulsifying capacity as a result of hydrolysis was observed [35].

### 3.3. Antioxidant Properties

The use of four methods to measure antioxidant activity allows for a more holistic determination of the total antioxidant capacity of a given compound or group of compounds, as there are some limitations for each analysis that may disturb the correct assessment of the antioxidant activity of a given sample if it were evaluated by only one analytical method [36, 37]. Among many methods used for evaluating antioxidant activity, the DPPH and ABTS tests are the most popular and commonly performed due to their simplicity, quickness, sensitivity, and usage of a stable radical and are very often carried out in tandem [38–40].

The ABTS and DPPH methods are based on both SET (single electron transfer) and HAT (hydrogen atom transfer) mechanisms, whereas the determination of antioxidant activity by the FRAP and reducing power measurement assays is based solely on the HAT mechanism [41].

**Figure 5:**
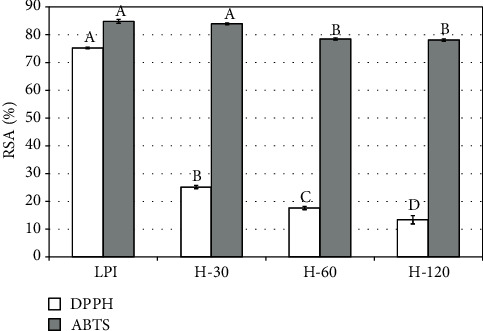
Antioxidant activity expressed as radical scavenging activity. The presented values are means ± standard deviations (*n* = 3). Values with different letters (a–d) in plot bars for the same method differ significantly at *P* ≤ 0.05.

**Figure 6:**
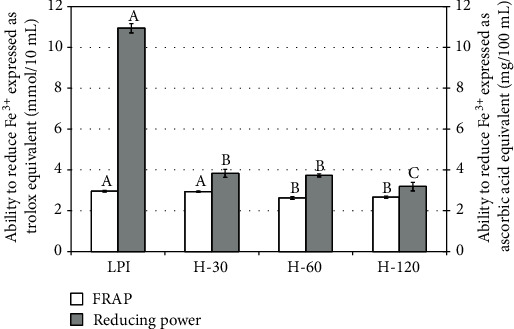
Antioxidant activity expressed as ability to reduce Fe^3+^. The presented values are means ± standard deviations (*n* = 3). Values with different letters (a–c) in plot bars for the same method differ significantly at *P* ≤ 0.05.

Analyses of antioxidant activity performed by four various methods showed that the results obtained were strongly dependent on the measurement methods (Figures 5 and 6). Large differences in antioxidant activity between the isolate and hydrolysates were noted for DPPH free radical scavenging and reducing power assays. The half-hour hydrolysis caused higher reduction in antioxidant activity of the product than extending the length of this process to 2 hours. With regard to the remaining two methods, i.e., using ABTS free radicals and FRAP, there were no such big differences. In these cases, a statistically significant reduction in antioxidant activity was noticed in the H-60 and H-120 hydrolysates. Guo et al. [42] reported that lupin protein isolate had lower antioxidant activity determined with DPPH and FRAP methods than hydrolysate obtained with Alcalase after 15 minutes of the process. Then, the antioxidant activity decreased with the subsequent slight increase at the end of the process. When compared to our study, differences may be due to the method of preparing the protein isolate; in our work, the flour was not defatted before the procedure of protein isolation. It could have caused migration of some amounts of fat together with antioxidant compounds into the isolate. Pena-Ramos and Xiong [43], who studied the effect of enzymatic hydrolysis of soy protein isolate on the antioxidant activity, found that hydrolysates obtained after a one-hour process with the use of Alcalase showed a little higher antioxidant activity than protein isolate and the remaining products of hydrolysis.

## 4. Conclusions

Changes in the length of polypeptide chains caused by hydrolysis and the accompanying significant decrease in surface hydrophobicity affected substantially the examined functional properties. This was manifested by the reduced emulsification capacity of hydrolysates and much poorer emulsion stability compared to LPI. The peptide chains formed as a result of proteolysis were too short, and hydrophobic domains were exposed insufficiently to create the film on the interface stable enough to limit coalescence.

On the other hand, it was observed that along with the prolongation of the hydrolysis time and the decrease in the protein average molecular weight, the foam capacity of hydrolysates was improved, although stability of the foam decreased.

In comparison with the hydrolysis products, LPI was characterized by much lower foam formation capabilities. The improvement of foaming properties due to two-hour proteolysis was connected with the presence of low-molecular *δ* conglutin, LMW subunits of vicilin-like *β* conglutins, and small subunits of *γ* conglutins.

When considering antioxidant properties, LPI was characterized by a distinctly better antioxidant activity than hydrolysates, as measured by the DPPH method and as reducing power, whereas the results obtained by the ABTS and FRAP methods were comparable.

## Figures and Tables

**Table 1:** Relative content (%) of protein fractions in lupin protein preparations (results of SDS-PAGE).

Molecular weight (kDa)	Protein content (%)				
	LPI	H-30	H-60	H-120	
>200	3.1	10.3	0.3	2.0	
74-100	1.6	0.0	0.0	0.0	
42-66	30.7	13.3	17.8	10.0	Acidic subunits of *α* conglutin, HMW subunits of *β* conglutin
34-40	16.0	0.0	0.0	0.0	IMW subunits of *β* conglutin
26-34	8.8	0.3	1.1	0.0	IMW subunits of *β* conglutin, large subunits of *γ*-conglutin
20-22	16.3	0.5	6.8	4.7	Basic subunits of *α* conglutin
18-20	6.4	1.3	0.0	0.0	LMW subunits of *β* conglutin
<15	17.0	74.4	74.0	83.3	Subunits of *δ* conglutin and products of hydrolysis

**Table 2:** Relative content (%) of protein fractions in lupin protein preparations (Schägger and von Jagow [19] procedure).

Molecular weight (kDa)	Protein content (%)			
	LPI	H-30	H-60	H-120
>100	2.5			
74-100	2.8			
43-65	26.9	31.2	27.1	28.8
34-40	8.0	14.7	6.0	0.0
24-33	20.3	9.2	27.7	25.5
20-23	4.9	8.9	11.8	11.1
18-20	7.7	0.6	0.0	0.0
12-16	16.8	9.0	5.2	3.1
<10	10.1	26.4	22.2	31.5

**Table 3:** Functional properties of LPI and its hydrolysates (H-30, H-60, and H-120).

	LPI	H-30	H-60	H-120
FC (%)	62.5 ± 0.9^a^	72.7 ± 2.0^b^	70.6 ± 1.3^b^	74.1 ± 2.0^b^
FO (mL)	153.4 ± 6.8^a^	46.0 ± 5.0^b^	33.4 ± 3.9^c^	28.5 ± 2.4^c^
LD_5_ (%)	66.2 ± 7.2^a^	79.9 ± 2.9^b^	81.0 ± 4.7^b^	81.8 ± 1.3^b^
EAI (m^2^/g)	10.9 ± 0.6^a^	8.5 ± 0.3^b^	7.3 ± 0.3^c^	7.2 ± 0.6^c^
ESI (min)	59.1 ± 7.0^a^	13.3 ± 2.2^b^	9.4 ± 1.1^b^	11.6 ± 2.2^b^

The presented values are means ± standard deviations (*n* = 3). Values with different superscripts (a–c) in rows differ significantly at *P* ≤ 0.05.

## Data Availability

The data used to support the findings of this study are available from the corresponding author upon request.
